# Behind the Scenes of Online Therapeutic Feedback in Blended Therapy for Depression: Mixed-Methods Observational Study

**DOI:** 10.2196/jmir.9890

**Published:** 2018-05-03

**Authors:** Mayke Mol, Els Dozeman, Simon Provoost, Anneke van Schaik, Heleen Riper, Johannes H Smit

**Affiliations:** ^1^ Department of Research and Innovation GGZ inGeest Specialized Mental Health Care Amsterdam Netherlands; ^2^ Department of Psychiatry Amsterdam Public Health Research Institute VU University Medical Center Amsterdam Netherlands; ^3^ Department of Clinical, Neuro and Developmental Psychology Clinical Psychology Section Vrije Universiteit Amsterdam and the Amsterdam Public Health Research Institute Amsterdam Netherlands; ^4^ Institute of Telepsychiatry University of Southern Denmark Odense Denmark

**Keywords:** cognitive behavioral therapy, eHealth, depressive disorder

## Abstract

**Background:**

In Internet-delivered cognitive behavioral therapies (iCBT), written feedback by therapists is a substantial part of therapy. However, it is not yet known how this feedback should be given best and which specific therapist behaviors and content are most beneficial for patients. General instructions for written feedback are available, but the uptake and effectiveness of these instructions in iCBT have not been studied yet.

**Objective:**

This study aimed to identify therapist behaviors in written online communication with patients in blended CBT for adult depression in routine secondary mental health care, to identify the extent to which the therapists adhere to feedback instructions, and to explore whether therapist behaviors and adherence to feedback instructions are associated with patient outcome.

**Methods:**

Adults receiving blended CBT (10 online sessions in combination with 5 face-to-face sessions) for depression in routine mental health care were recruited in the context of the European implementation project MasterMind. A qualitative content analysis was used to identify therapist behaviors in online written feedback messages, and a checklist for the feedback instruction adherence of the therapists was developed. Correlations were explored between the therapist behaviors, therapist instruction adherence, and patient outcomes (number of completed online sessions and symptom change scores).

**Results:**

A total of 45 patients (73%, 33/45 female, mean age 35.9 years) received 219 feedback messages given by 19 therapists (84%, 16/19 female). The most frequently used therapist behaviors were informing, encouraging, and affirming. However, these were not related to patient outcomes. Although infrequently used, confronting was positively correlated with session completion (ρ=.342, *P*=.02). Therapists adhered to most of the feedback instructions. Only 2 feedback aspects were correlated with session completion: the more therapists adhere to instructions containing structure (limiting to 2 subjects and sending feedback within 3 working days) and readability (short sentences and short paragraphs), the less online sessions were completed (ρ=−.340, *P*=.02 and ρ=−.361, *P*=.02, respectively). No associations were found with depression symptom change scores.

**Conclusions:**

The therapist behaviors found in this study are comparable to previous research. The findings suggest that online feedback instructions for therapists provide sufficient guidance to communicate in a supportive and positive manner with patients. However, the instructions might be improved by adding more *therapeutic* techniques besides the focus on style and form.

## Introduction

### Internet-Delivered Cognitive Behavioral Therapy

There is considerable evidence that Internet-based interventions are effective for the treatment of mild, moderate, and major depression [1-3]. Therapist-guided, Internet-delivered cognitive behavioral therapy (iCBT) has been found to be more effective than unguided iCBT [1,4] and has also been found to be equally effective compared with face-to-face-delivered CBT [5]. Beyond these findings, a number of studies focused on nonspecific factors that might be effective in iCBT (eg, therapeutic alliance, therapist competence, and placebo-expectancy effects) and especially showed interest in the role of therapist guidance in iCBT [6]. So far, mixed results have been found. In a systematic review, Richards and Richardson, eg, found that the way guidance is given has an impact on treatment adherence in depressed patients [4]. Therapist-guided iCBT had a 72% completion rate, iCBT interventions with administrative support (support by staff to guide patients through the program in a nontherapeutic way) 65%, and interventions with no support at all 26%. For Internet-delivered problem-solving treatment (PST), there also is evidence that the level of support is important in reaching effects for patients with depression [7]. Patients who received PST with weekly support from a coach improved significantly more than the waitlist control group. In the group that received no support, completion rates were lowest (22%), and the completion rates were highest in the group that received nonspecific support (60%). Patients who received weekly support had comparable completion rates with patients who received “support on request” (33% and 31%, respectively). In a study by Titov et al, patients with depression showed significant clinical improvement after receiving iCBT, regardless of whether the support came from a therapist or a technician [8]. Findings of a recent American study indicate that iCBT with 5 hours of therapeutic face-to-face contact was noninferior to CBT that provided over 8 additional hours of therapist contact for patients with depression [9].

### Therapist Behaviors

However, there is much more to discover about online guidance. One point of interest is how therapists give online feedback to their patients. This can be done by looking at the communication strategies and content they use in their written support. For example, looking at therapist behaviors such as validating what patients write (eg, “That must be very difficult for you...”) and stimulating patients to come up with their own solution (eg, “When was the last time you felt that way? What did you think and what did you do differently?”). Written feedback is a substantial part of Internet-based treatments and requires specific skills of therapists. It is therefore interesting to further explore such therapeutic microprocesses in online feedback because this part of therapy may be very relevant in the adherence and also the effectiveness of iCBT [10].

### Therapist Behaviors in Face-to-Face Therapy

The content of feedback and its impact on treatment results have been studied in face-to-face–delivered psychotherapies, and especially in CBT. Studies have identified different therapist behaviors that are frequently used in CBT sessions with patients. These behaviors range from expressing empathy, making supportive communications (eg, encourage, praise, or guide the patient), asking directive questions, and confronting patients with different points of view [11-13]. Self-disclosures by therapists appear to be infrequently used [11], although these are generally considered helpful by patients in the therapeutic process [14,15]. In addition, research shows that therapist behaviors such as expressing empathy, giving positive regards, and confronting patients can have a positive impact on treatment outcome in CBT for various patients such as people with depression [11].

### Therapist Behaviors in Internet-Delivered Cognitive Behavioral Therapy

Therapist behaviors in iCBT have also been studied. This was done for several psychiatric diseases such as eating disorders [16], insomnia [17], anxiety [18], and depression [19,20]. Comparable to the behaviors in face-to-face therapies, the most frequently used therapist behaviors were encouraging, reinforcing, and supporting patients. When looking at the association between these therapist behaviors, patient treatment outcome, and patient online session completion, mixed results were found. Holländare et al found that encouraging, guiding, and affirming were strongly associated with session completion [19]. Encouraging, affirming, and self-disclosure were weakly to moderately associated with an improvement in depressive symptoms. The most important finding by Paxling et al was the effect of therapists’ task reinforcement (eg, reinforcing completed assignments) on session completion as well as treatment outcome [18]. Interestingly, a negative association was found between deadline flexibility of therapists and treatment outcome. Thus, the more lenient therapists were with homework assignment deadlines, the fewer patients improved. In a replication study of Schneider et al, the same type of therapist behaviors were found with the addition of a few more behavior categories (eg, asking questions) [20]. However, a different distribution of the therapist behavior frequencies was found, and the outcomes were different for patients with depression than for patients with anxiety. Thus, the way online feedback is provided by therapists differs across studies, patients, interventions, and possibly also the instructions used for feedback.

### Online Feedback Instructions

In addition to more general communicative behaviors of therapists, the extent to which they follow instructions for online feedback may also influence treatment effectiveness. Research on written feedback predominantly stems from the field of education. Some of the main principles can be applied to online therapeutic feedback as well. Overall, research shows that effective written feedback is timely (provided in time), selective (commenting only on 2 or 3 things that someone can change), balanced (pointing out positive aspects as well as areas in need of improvement), forward-looking (suggesting how to improve), and understandable (written in a language that someone will understand) [21]. Instructions for training therapists in written feedback are adapted to the therapeutic process but also comparable to those used in education (eg, beginning with a compliment, responding within 3 working days, or being careful with giving solutions). The elements in these instructions are primarily based on expert opinion rather than theory and mainly aim to motivate and support the patients, respond to the content of homework, and structure the feedback.

### Study Objectives

In this study, written feedback will be studied in blended CBT, in the context of the European implementation project MasterMind [22-24], with a focus on therapist behaviors and on the extent to which feedback instructions are followed. In the Netherlands, iCBT for depression is slowly but increasingly adopted in routine mental health care mostly in a blended format. Blended CBT entails one integrated, standardized CBT treatment protocol that combines face-to-face sessions and digital modules to the best clinical benefit for patients and therapists [25]. The evidence of blended CBT over iCBT is unfortunately still scarce. Some first studies indicate that potential benefits of blended CBT are saving therapist time without reducing therapeutic outcome, lower treatment dropout rates, more emphasis on patient self-management, more face-to-face therapy time for deepening the CBT elements, and targeting another (often more complicated) population than iCBT [6,9,26]. The objectives of this study were to (1) identify therapist behaviors in written online communication from therapists to patients in blended CBT for adult depression in routine secondary mental health care, (2) identify the extent to which therapists adhere to feedback instructions, and (3) investigate whether therapist behaviors and therapist adherence to feedback instructions are associated with patient outcome (symptom change scores and number of completed online sessions).

## Methods

### Design

For the purpose of this observational study, the feedback messages of 45 Dutch patients that were offered blended CBT for depression by 19 therapists in routine mental health care were recruited between April 2015 and February 2017 from one outpatient clinic. This clinic was one of the participating MasterMind sites and was selected for this study because it offered a blended treatment protocol to patients within secondary health care, and the online usage information was made available for research. Patients received 219 feedback messages through a secure Web-based platform [27]. A mixed-method design was chosen to explore the content of the feedback messages: a directed qualitative content analysis [28] was used to identify therapist behaviors, and a checklist for the feedback instruction adherence of therapists was developed. To explore correlations between the frequency of therapist behaviors, scores on the checklist, and patients’ outcomes, an explorative quantitative approach was used.

The study was approved by a Medical Ethics Committee. They confirmed that the “Medical Research Involving Human Subjects Act” does not apply (registration number 2014.580) because the patients in this study are not required to follow certain procedures on behalf of the research (no randomization) and routine practice was followed. An internal scientific research committee approved the research proposal (CWO 2015-005).

### Participants

#### Patients

Patients were recruited through their therapists. Eligible patients received study information and an information leaflet from their therapist. After approval for telephone contact with researchers for additional information, patients received an informed consent. Patients were invited for participation in MasterMind if they (1) were aged 18 years or older; (2) had a mild, moderate, or severe depression as a primary diagnosis according to the therapist; and (3) were indicated for cognitive behavioral treatment for depression following routine secondary mental health care procedures. All patients needed to explicitly consent to take part in the study. Patients were excluded from the study if they (1) did not have a valid email address and did not have a computer with Internet access and (2) did not have adequate Dutch language skills (both verbal and written).

#### Therapists

Therapists who were trained in iCBT or who were motived for iCBT were invited to participate in the MasterMind study. They were recruited through team managers and eHealth attention officers of the different therapist teams. The iCBT training consisted of a 4-hour group training, provided by the outpatient clinic. During the training, the functionalities on the online platform were shown, and therapists got the chance to practice with a fictional patient. The therapists received individual instructions, access to the blended CBT treatment protocol online, and the feedback instructions. In addition, monthly 1-hour group sessions were organized where the therapists could exchange their experiences with each other.

The feedback instructions for therapists comprised general and specific elements that go in to the structure of the messages (eg, correct greeting, limiting to 2 subjects), readability (short sentences and paragraphs), writing style (eg, limiting abbreviations and misspellings, use of emoticons), referring to parts of the treatment (eg, filling in the diary, referring to the next online session), and communication skills (eg summarizing, not providing solutions).

#### Intervention

In the blended CBT treatment for depression of the outpatient clinic, it was agreed upon in advance that patients would receive 10 sessions online and meet with their therapist in 5 face-to-face sessions biweekly. In practice, therapists could deviate from the protocol by repeating online sessions. The online and individual face-to-face sessions were based on evidence-based treatment protocols for face-to-face CBT and are in agreement with multidisciplinary instructions for depression [29]. There were 4 core components: (1) psychoeducation, (2) cognitive restructuring, (3) behavioral activation, and (4) relapse prevention. Besides the online sessions on the treatment platform, patients were given online access to a diary and filled out questionnaires to monitor their symptoms. After each completed online session, the therapist (the same therapist as in the face-to-face sessions) wrote a feedback message to the patient. Patient and therapist could additionally communicate through a message function about practical issues (eg, about upcoming appointments and reminders or questions about assignments).

### Measures

Patient information on selected demographics (eg, age, gender, employment status) and clinical data (eg, use of medication) were obtained by an online self-report questionnaire at baseline. Demographic and background information (eg, treatment and iCBT experience) of the therapists were obtained by an online self-report questionnaire at the end of the study. Usage information (eg, number of online sessions followed and number of feedback messages) was obtained from the online platform.

Session completion was defined as the number of completed online sessions per patient. Symptom improvement was measured with the 16-item Quick Inventory of Depressive Symptomatology (QIDS) [30]. The total score varies from 0 to 27, with higher scores being indicative of a higher severity of depressive symptoms. The QIDS was administered weekly on the online platform during the course of the treatment. The number of QIDS measures can vary, with up to 30 weekly measures. Of each patient, the baseline scores were included, and the last known value was used as a posttreatment score. The change score on the QIDS was calculated by subtracting the baseline measurement from the final measurement.

### Coding of Therapist Behavior and Adherence

To subtract therapist behaviors from the 219 online feedback messages, a coding matrix was developed, with 9 main categories and 13 subcategories (see Multimedia Appendix 1). The coding categories were based on the directed content analysis; categories from prior research [18,19,31] were used to develop the initial coding scheme before analyzing the data [25]. To score the therapists’ adherence to the feedback instructions, a coding checklist was created based on the instructions that the therapists received. In total, there were 6 main categories and 19 different subcategories with a dichotomous scale (present or not present, see Multimedia Appendix 2).

The coding matrix and checklist were first tested by researcher ED by coding 4 feedback messages from 2 randomly selected patients. Each of the included feedback messages was then anonymously coded and scored by researchers MM and SP. For the coding of therapist behaviors, qualitative data analysis software, ATLAS.ti 7.5.18 (ATLAS.ti Scientific Software Development GmBH, Berlin, Germany), was used.

To investigate interrater reliability, both researchers (MM and SP) coded 60 transcripts of therapeutic feedback from 10 randomly selected patients. The intraclass correlation coefficient for the therapist behaviors was .83 (95% CI 0.82-0.85) indicating good interrater reliability, based on 2-way mixed-effects agreement model [32,33]. The interrater reliability for the feedback instruction adherence categories was found to be kappa (κ)=.84 (*P*<.001, 95% CI 0.80-0.87), indicating a good agreement between the raters as well. After reaching agreement, the remaining messages (n=159) were equally divided between the 2 coders. As analysis proceeded, additional codes were developed, and the initial coding matrix and checklist were revised, discussed, and refined.

The total frequency of therapist behaviors was calculated with a query tool in ATLAS.ti. A frequency score represented the total number of times the therapist displayed a behavior in the feedback messages sent to the patient (eg, total number of informing the patient about the assignments). To correct for the number of received feedback messages (eg, some patients received 4 messages, and the others received 8 messages), relative frequencies were used (frequency of one category divided by the total number of frequencies of all categories per patient). The percentage of therapists’ adherence to the instructions for each patient was calculated by the frequency of the adherence (eg, the total number of times a therapist started with giving a compliment) divided by the total number of messages received by a patient.

### Analyses

Statistics were conducted using IBM SPSS (SPSS Inc., Chicago IL), version 22. First, descriptive statistics (means, SDs, percentages) were used to describe the patient and therapist sample, number of online sessions, and symptom improvement. Descriptive statistics were then used to examine the frequencies of therapist behaviors and percentages of therapist instruction adherence in the messages to the patients. Spearman correlation analyses, 2-sided, were conducted to assess the relationship between the therapist behaviors, feedback adherence scores and session completion, and symptom improvement. Spearman rho was used to avoid violation of assumptions of normality. Due to the small sample size, only explorative analysis, no missing values imputation techniques and no post-hoc correction for multiple testing (ie, Bonferroni), were applied.

## Results

### Patients’ and Therapists’ Characteristics

A total of 45 patients (73%, 33/45 female, mean age 35.9 years) were given blended CBT in routine care by 19 therapists. Patients’ characteristics can be found in Table 1. Of the 19 therapists (84%, 16/19 female), most were licensed psychologists (53%, 10/19), others were psychologists in training under supervision for health care psychologists (26%, 5/19) or mental health nurses (21%, 4/19). Moreover, 11% (2/19) of the therapists had less than 3 years of professional experience, 26% (5/19) had between 3 and 5 years of experience, 37% (7/19) had between 5 and 10 years of experience, and 21% (4/19) had more than 10 years of experience. The experience with iCBT treatments varied among the therapists: 32% (6/19) had given less than 5 iCBT treatments, 26% (5/19) had given between 5 and 10 treatments, 21% (4/19) had given between 10 and 15 treatments, and 16% (3/19) had given more than 15 treatments.

**Table 1:** Characteristics of patients at baseline.

Patients’ characteristics		Statistics (n=45)
Gender, female, n (%)		33 (73)
Age in years, mean (SD; range)		35.9 (12.3; 21-64)
**Education level, n (%)**		
	Secondary education level	15 (37)
	Higher education level	25 (61)
Employment, yes, n (%)		21 (51)
Antidepressant use, yes, n (%)		10 (24)
**Duration of current depression symptoms, n (%)**		
	Duration of current depression symptoms less than 3 months	8 (20)
	Duration of current depression symptoms between 3 and 12 months	22 (54)
	Duration of current depression symptoms more than 1 year	10 (24)

### Frequencies of Therapist Behaviors and Percentages of Therapist Instruction Adherence

Multimedia Appendix 1 lists the categories, definitions, and examples of the therapist behaviors. In total, 1825 therapist behaviors were coded. The most frequently used therapist behaviors were informing (27.56%, 503/1825; eg, informing the patient about the next session or specific assignments), encouraging (23.56%, 430/1825; eg, praising past behavior), and affirming (22.25%, 406/1825; eg, normalizing behavior, summarizing what the patient has written or said). Making self-disclosures, confronting, and emphasizing the responsibility of the patient were never or rarely used.

An overview of the percentages of the categories, descriptions, and examples of adherence to the feedback instructions can be found in Multimedia Appendix 2. The therapists adhered in most cases to correct greeting and ending of messages (95.9%, 210/219). They also scored high on writing style (93.6%, 205/219; eg, limiting of abbreviations and misspellings) and structure (87.7%, 192/219; eg, limiting to 2 subjects and sending the feedback within 3 working days). Therapists scored the lowest on referring (34%, 74.5/219; eg, referring to monitoring of the symptoms or reflecting on the dairy). Within the category communication skills, therapists were very often careful with giving solutions (95.6%, 209/219) and regularly showed in their writing that they read the patients’ homework (88.6%, 194/219). Formulating sentences as hypotheses is something the therapists did not often apply (10.5%, 23/219).

### Session Completion and Symptom Improvement

The 45 patients completed, on average, 6.3 online sessions (Table 2). On average, patients received 4.9 feedback messages (SD 2.7; range 1-10). One feedback message contained an average of 139 words (SD 95.4; range 1-504), 14.2 words in one sentence (SD 4.0; range 1-26), 3.5 sentences in a paragraph (SD 1.9; 1-17), and 2.9 paragraphs (SD 1.6; 1-10).

From 7 patients, all QIDS data were missing because their therapists did not activate the online monitoring, leaving 38 patients for this exploration. Results on depressive symptoms showed that at baseline, the patients scored, on average, 15.8 points (SD 3.8) on the QIDS, and at postmeasurement, the patients scored, on average, 11.0 points (SD 6.0), so there was an average reduction of 4.8 points (SD 6.4). Looking at symptom severity at baseline, 8% (3/38) of the patients had mild symptoms, 34% (13/38) had moderate symptoms, and 58% (22/38) had (very) severe symptoms (Table 3). At posttreatment, 21% (8/38) of the patients had no symptoms, 29% (11/38) had mild symptoms, 24% (9/38) had moderate symptoms, and 26% (10/38) had (very) severe symptoms. In total, 63% (24/38) of the patients improved on one or more categories (Table 4). Moreover, 24% (9/38) of the patients showed no change, and 13% (5/38) deteriorated in a category.

Multimedia Appendix 3 contains case descriptions of 3 patients.

### Correlations of Therapist Behaviors With Session Completion and Symptom Improvement

One correlation between therapist behaviors and session completion was found (Table 5): the therapist behavior confronting was positively correlated with online session completion (ρ=.342, *P*=.02). This indicates that more confrontations were related to completing more online sessions. No significant correlations were found with symptom improvement.

### Correlations of Therapist Instruction Adherence With Session Completion and Symptom Improvement

In Table 6, correlations of therapist instruction adherence with session completion and symptom improvement are shown. Statistically significant negative medium correlations were found between therapist instruction adherence and completed online sessions for structure (ρ=−.340, *P*=.02) and readability (ρ=−.361, *P*=.02). Meaning that the more therapists adhered to instructions containing structure (limiting to 2 subjects and sending feedback within 3 working days) and readability (short sentences and short paragraphs), the less online sessions were completed. No significant correlations were found with symptom improvement.

**Table 2:** Treatment completion and duration (n=45).

Treatment completion and duration	Mean (SD; range)
Completed online sessions,	6.3 (2.6; 2-11)
Completed face-to-face sessions	7.1 (2.7; 2-13)
Completed face-to-face + online sessions	13.4 (4.4; 5-23)
Treatment duration in weeks	26.2 (11.2; 8-52)
Period of online activity in weeks	17.8 (10.9; 2-45)

**Table 3:** Severity Quick Inventory of Depressive Symptomatology scores at baseline and postmeasurement.

Severity Quick Inventory of Depressive Symptomatology	Quick Inventory of Depressive Symptomatology (n=38), n (%)	
	Baseline	Postmeasurement
None	0 (0)	8 (21)
Mild	3 (8)	11 (29)
Moderate	13 (34)	9 (24)
Severe	20 (53)	5 (13)
Very severe	2 (5)	5 (13)

**Table 4:** Changes in symptom severity (n=38).

Change in depressive symptom severity	n (%)
Reduction in 1 category	11 (29)
Reduction in 2 categories	10 (26)
Reduction in 3 categories	3 (8)
Deterioration	5 (13)
No change	9 (24)

**Table 5:** Correlations of therapist behaviors with session completion and symptom improvement.

Therapist behavior	Session completion (n=45)	Change score Quick Inventory of Depressive Symptomatology (n=38)
Emphasizing responsibility	.094	.278
Affirming	.074	.035
Clarifying the framework	.232	.069
Self-disclosure	—^a^	—
Informing	−.087	−.249
Confronting	.342^b^	.184
Urging	.258	.310
Encouraging	−.054	−.008
Guiding	−.055	.146
Questions	.066	.115

^a^Indicates "not applicable"; self-disclosures did not occur.

^b^*P*<.05, a positive correlation indicates more session completion.

**Table 6:** Correlations of therapist instruction adherence with session completion and symptom improvement.

Therapist instruction adherence	Session completion (n=45)	Change score Quick Inventory of Depressive Symptomatology (n=38)
Greeting and ending	−.277	−.064
Communication skills	−.146	−.212
Structure	−.340^a^	−.214
Referring	.170	−.085
Readability	−.361^a^	−.185
Writing style	−.150	−.139

^a^*P*<.05, a positive correlation indicates more session completion.

## Discussion

### Aim of This Study

This observational study has uncovered several important factors in the content of online feedback messages in blended iCBT for depression. We further explored therapist behaviors and the extent to which therapists wrote their feedback according to their instructions. In addition, we wanted to know if therapist behaviors and adherence to the feedback instructions could be linked to patient adherence and treatment outcome. The study was carried out in a Dutch sample of participants of the MasterMind study, in routine practice, in a patient population with mild to (very) severe depressive symptoms and with a diverse group of trained and skilled therapists.

### Principal Findings

Results show that therapist behaviors in relation to the online guidance are informing the patient about the functionalities on the platform, encouraging the patient by praising past behavior or inciting future behavior, and affirming by showing interest in the thoughts, emotions, and behaviors of the patient. Making self-disclosures, confronting, and emphasizing the responsibility of the patient are never or infrequently used. This is largely in line with the frequencies of the categories found by Holländare et al and may indicate that therapists use the same CBT principles in their written communication as in their face-to-face communication with the patient [19]. Previous research also found that more supportive therapists’ behaviors are used frequently in iCBT and that behaviors such as confronting and self-disclosures are seldom used [18,28]. However, in contrast to the findings of Holländare et al, we found that one and also a different therapist behavior correlated with module completion, and we also found that none of the therapist behaviors were related to symptom improvement. A possible explanation for this difference can be found in the patient group; in Holländare et al’s study, patients with partially remitted depression were included within the context of a randomized controlled trial.

Although therapists applied confronting in limited cases (<1%), this was positively correlated with online session completion. In face-to-face CBT, the occurrence of confrontations has been found to be somewhat higher (6%-14.3%), but is also significantly correlated with therapy outcomes [11]. Hill et al argued that “confrontation often interrupts the client’s thinking by presenting discrepancies and another point of view […]. Although confrontation feels negative at the time, such disruption may be a necessary foundation for change” [15].

Furthermore, therapists followed the feedback instructions that were used in this study on most of the defined elements, such as beginning with a compliment and being careful about providing solutions too soon. Different than expected, only half of the therapists formulated their sentences as hypotheses, and did so in only 10% of the feedback messages (eg, “It sounds like you are not sure, is that correct?”). Misspellings occurred regularly: in 21.5% of the feedback messages, therapists made more than 3 spelling mistakes. One of the possible explanations for this is that the treatment platform did not contain a spelling corrector, and it may have taken therapists more time to correct their own writing. Emoticons were not used often, as only 3 therapists sometimes used an (positive) emoticon. In the “Supportive Accountability” model by Mohr and Cuijpers, it is argued that therapists may mirror the content, style, tense, and cues (eg, emoticons) in online communication by patients to create mutual trust [31]. In this model, it is also pointed out that people pay attention to the timing and date stamps of the responses. This means responses should be timely because delays may be perceived as expressing lack of affection. In our study, the therapists sent their feedback within the limit of 3 working days in almost 80% of the cases.

Only negative associations were found with therapist instruction adherence and session completion. Providing structure and the readability was significantly negatively associated with session completion. This means that if the therapists adhered more to writing short sentences and paragraphs and the more they limited their feedback to 2 different subjects and sent the feedback back within 3 working days, the less online sessions were completed. These findings might be explained by the adaptive, and also reactive, style of the therapists to the behavior of the patient. When patients are doing well on the online platform, they are more flexible with certain elements of the instructions. On the other hand, when patients display more difficulties or when the therapist gets the feeling that he or she is losing contact with the patient, therapists may be more inclined to adhere more to some parts of the instructions. Schneider et al also found that therapists were responsive in their online feedback and that they increased some behaviors during the course of treatment when patient depressive symptoms worsened. There are similar indications in psychotherapy, where more flexibility of therapists was found related to better treatment outcomes than therapists who were less flexible [34].

### Strengths and Limitations

The study took place in a naturalistic setting, with routine care patients and therapists and without the restrictions of a randomized controlled trial. Patient demographic characteristics in the study sample are comparable to blended CBT research, also in routine care [9,26]. Previous studies were carried out in small samples of therapists (3-5), often trained students, who delivered treatment in a research setting [16-19]. With the use of a directed approach of the content analysis, the findings of the previous research were supported and extended. We found the same proportions of categories as Holländare et al with the addition of the category “asking questions” [19]. This was also found by Schneider et al when they replicated the study conducted by Paxling et al [18,20].

In addition, there are several limitations to this study. The generalizability of the results is limited because of the small sample size. With a greater sample size, it would have been possible to explore initial symptom severity as a predictor of the use of different therapist behaviors. The exploration of this association would be interesting for further research. Second, although this study was able to capture a group of experienced professionals, the distribution of patients over the therapist was slightly skewed. Half of the therapists treated 3 to 6 patients, and the other half treated 1 or 2 patients. Due to the small sample size, it was not possible to explore potential differences in writing style or skills between the therapists. Furthermore, in face-to-face treatment, therapist characteristics such as age, gender, and ethnicity of the therapist seemed not to be related to patient treatment outcomes [35], but therapist facilitative interpersonal skills were found to be a successful predictor of treatment outcome [1]. To further explore therapist online feedback, it would be interesting to look at therapeutic skills as well. In this study, there was a high variability in the number of words in the feedback messages, and it could also be interesting to further explore this. Third, within this observational study, only prepost data and explorative analyses and, no post-hoc corrections, were used. The found correlations should be interpreted with care. Previous research showed that it is possible that therapist behaviors change over the course of treatment, with more focus on certain categories at the beginning of treatment versus the end of treatment [19,20]. Finally, this study only focused on the online part of the blended treatment and not on the content in the face-to-face sessions.

### Conclusions

In sum, this study showed that in blended CBT for depression, therapists primarily used supportive and positive communications like informing, encouraging, and affirming patient behavior. Therapists refrained from using therapeutic techniques, such as making self-disclosures, urging, and confronting. This can be explained by the way the online feedback instructions were constructed. They provided the therapists guidelines that concentrate on style and form instructions, and this is also reflected in the adherence of the therapists to most of these instructions. It can be suggested that the instructions should also focus more on “disruptive” therapeutic techniques that can foster patients to address their symptoms. The blended format can give the therapist more flexibility in writing feedback because of the combination with face-to-face contact, meaning that therapists can check the interpretation of their online feedback with the patients in the face-to-face sessions. The combination with online contact gives the therapist the possibility to incorporate elements and reflect on issues that were discussed in the face-to-face sessions. On the other hand, therapists are aware that online communications can emotionally positively and also negatively affect the patient, without them being there, and are therefore careful in their communications. The therapists may miss nonverbal cues such as facial expressions and are not able to respond immediately. Writing feedback requires the therapist to assess whether the patient can correctly understand it. The extent to which this calls for specific competencies of the “online” therapist is assumed and requires further exploration. Additional research is needed to further explore the content of online feedback. With an experimental design, more causal explanations can, eg, be made about the amount of certain therapist behaviors, the interaction with the written content of the patients, patient expectations or the timing of feedback, and also the interaction with the contact of the face-to-face sessions. With more knowledge, instructions on feedback can be enriched, and therapists can be offered more guidance in giving feedback.

## Appendix

Multimedia Appendix 1 Main and subcategories therapist behaviors, definitions, examples and percentages out of 219 feedback messages.

Multimedia Appendix 2 Main and feedback instructions, definitions, examples and percentages out of 219 feedback messages.

Multimedia Appendix 3 Case descriptions of 3 patients.

## Abbreviations

CBT: cognitive behavioral therapy
iCBT: Internet-delivered cognitive behavioral therapy
PST: problem-solving treatment
QIDS: Quick Inventory of Depressive Symptomatology
