# Supplementary material for: Behind the Scenes of Online Therapeutic Feedback in Blended Therapy for Depression: Mixed-Methods Observational Study
Source: J Med Internet Res. 2018 May 3;20(5):e174. doi: 10.2196/jmir.9890 (PMC5958280; doi:10.2196/jmir.9890)
Supplement: Multimedia Appendix 1 [file jmir_v20i5e174_app1.pdf]

Multimedia Appendix 1. Main[a] and subcategories therapist behaviours, definitions, examples and percentages out of 219 feedback messages.

Category therapist behaviors	Definition	Example	%
**Emphasizing responsibility**	Emphasize the responsibility of the pt[b]	*It is useful for yourself to get insight in this by filling in the diary*	**0.1**
**Affirming**	Give attention/recognize/show interest in thoughts, emotions and behaviors of the pt and to consider them valid		**22.2**
Validating and interpreting	Confirm by interpreting and validating what the pt has written	*That must be very difficult for you I see that you are struggling with it*	10.9
Normalizing	Confirm by stating that the behavior of the pt often occurs/is normal	*It is not easy to .... /It is very common that you feel this way*	1.4
Summarizing online	Confirm by summarizing and repeating what the pt has written	*You write that you..../You are able to focus and enjoy your activities*	7.4
Summarizing f2f	Confirm by summarizing and repeating what the pt has said	*This morning we discussed that you....*	2.5
**Clarifying the framework**	Clarify/emphasize/remind the pt of the protocol/framework and give practical information	*You gave now finished the first part of the treatment, in the second part we will focus on....*	**3.9**
**Self-disclosure**	Use experiences and personal examples from the therapist's life	*This exercise has helped me with my sleeping problems*	**0**
**Informing**	Inform or refer to different functionalities on the online platform		**27.5**
Informing about the next session	Inform or refer to the next session and/or its content	*You can now proceed with session 5: Structure and planning*	10.4
Informing about the diary	Inform or refer to the diary and/or its content	*It is helpful to set a reminder in your phone to fill in the dairy everyday*	2.8
Informing about the monitoring	Inform or refer to the monitoring/ questionnaire and/or its content	*Your score on the QIDS is 7, so your symptoms are mild now*	1.5
Informing about the assignments	Inform or refer to the assignments in the previous session and/or its content	*You kept track of your activities during the weekend, I'm curious about your week-days*	3.7
Informing about the f2f	Inform or refer to face-to-face	*See you next week, at 14.00!*	9.1

session	session		
**Confronting**	Express a different opinion or disagree with the pt	*I do not think that your activities are useless, but I think the opposite!*	**0.4**
**Urging**	Urge to let the pt do something	*It is important that you try this*	**3.9**
**Encouraging**	Encourage/motivate past and future behavior of the pt		**23.4**
Praising past behavior	Praise something the pt has done in the past	*Even though you found it difficult, you did it, very good!*	14.5
Inciting future behavior	Incite something that the pt is planning to do	*Continue to keep up this good work! Good luck!*	8.9
**Guiding**	Give advice, information or suggestions		**11.3**
Psychoeducation	Give information on psychological processes	*The fact that it doesn't immediately give more pleasure is something we see often in a depression*	5.5
Giving suggestions	Give suggestions about alternative behavior/provide advice on how it can be addressed	*Try to think of something that's easy to do and cannot easily go wrong*	5.8
**Questions**	Ask or answer questions		**6.9**
Asking questions to clarify	Ask questions to better understand the behavior or emotions of the pt	*Is that something you already do?/ … is that correct?*	3.4
Asking 'thought' questions	Ask questions to encourage the pt to think further	*How would you like to feel and what should your life look like?*	3.2
Answering questions	Respond to questions of the pt	*Yes, I will repeat this session for you, so that you can practice more*	0.3

[a]Main categories are in bold.

[b]Pt stands for patient.
