# Supplementary material for: Behind the Scenes of Online Therapeutic Feedback in Blended Therapy for Depression: Mixed-Methods Observational Study
Source: J Med Internet Res. 2018 May 3;20(5):e174. doi: 10.2196/jmir.9890 (PMC5958280; doi:10.2196/jmir.9890)
Supplement: Multimedia Appendix 2 [file jmir_v20i5e174_app2.pdf]

Multimedia Appendix 2. Main[a] and subcategories feedback instructions, descriptions, examples and percentages out of 219 feedback messages.

Category feedback instructions	Description in instructions	Example	Yes %
**Greeting/ending**			**95.9**
Correct greeting	Use a greeting	*Dear [name pt[b]]*	95.4
Correct ending	Use a correct ending	*Good luck with the next session! Greetings, [name therapist]*	96.3
**Communication skills**			**69.4**
Begin with compliment	Begin the message with a compliment/positive approach	*From your answers I can see it was difficult, but you did very good!*	79.0
Summarize	Summarize the assignments of the pt, use his/her own words	*You clearly described your depression, the loss of interest/energy, negative thoughts and worrying.*	73.1
Reading homework	Show that you have read the homework by using their examples	*I see that you.../You say that you..../If I understand you correctly...*	88.6
Hypotheses	Formulate sentences as hypotheses	*That sounds like..... , is that correct?.. If I understand you correctly.....*	10.5
Giving no solutions	Give no solutions, let patients think of their own solutions. You can give them a starter in the right direction	*When was the last time you felt that way? What did you think and what did you do differently then?*	95.6
**Structure**			**87.7**
2 subjects	Give feedback on max two subjects in order to keep the structure clear		95.4
Within 3 working days	Give feedback within three working days. Make an agreement when the pt should finish the session and when you as a therapist can write the feedback		79.9
**Referring**			**34.0**
Referring diary	Reflect on the diary or when the pt did not succeed yet, address (again) the utility of the diary	*Good that you used the diary. I can see that your mood is different in the mornings than in the evenings, it that something you also experience?*	26.9
Referring monitoring	Refer to the monitoring questionnaire or when the pt did not succeed yet, ask to fill this in next time	*Your score on the QIDS is 7, so right now your symptoms are mild.*	11.4
Referring next session	End the message with a reference to the content of the next session	*I will open the next session for you. There you can further practice with the schemas*	41.6
Referring next f2f session	Confirm the next f2f appointment	*We will see each other again on May 19.*	56.2
**Readability**			**68.0**
Short sentences	Write short, clear sentences.		61.6
Short paragraphs	Write short, clear paragraphs		74.4
**Writing style**			**93.6**
Limit abbreviations	Limit your abbreviations		97.3

Limit misspellings	Be aware of misspellings		78.5
Limit emphasis	Limit text in caps, exclamation marks, underline text or make text bold		98.6
Correct emoticons	Use emoticons only when you know the meaning is clear for the pt and use it as a complement and not as a substation	*And do not forget the fun activities* □	100

[a]Main categories are in bold.

[b]Pt stands for patient.
