# Supplementary material for: Behind the Scenes of Online Therapeutic Feedback in Blended Therapy for Depression: Mixed-Methods Observational Study
Source: J Med Internet Res. 2018 May 3;20(5):e174. doi: 10.2196/jmir.9890 (PMC5958280; doi:10.2196/jmir.9890)
Supplement: Multimedia Appendix 3 [file jmir_v20i5e174_app3.pdf]

Multimedia Appendix 3. Case descriptions of three patients

Box 1. Case description of patient K.

Background information

K. is a 40 year old father of one child. He works as a chef. He suffers from fatigue, feelings of exhaustion, anhedonia, emotional instability and excessive guilt. His symptoms seem to be caused by an accumulation of stressors over the last few years (high workload, illness of his mother, job change of partner and worrying about his daughter), in combination with a habit of making high demands on himself and a need to always be strong. This is the third time he is experiencing these symptoms. There seems to be an overload of stress for some time now, which has now led to the development of a depressive disorder.

Treatment course

K. completed 11 online sessions (repeated online session 3) and had 5 face-to-face sessions with his therapist. At the beginning of treatment K. scores 17 on the QIDS, which is considered severe. The treatment was successful, after 15 weeks of treatment the depression was in remission (QIDS score 7, severity mild). Most used therapist behaviors were Affirming (relative frequency 24.7), Informing (relative frequency 19.7) and Encouraging (relative frequency 18,3).

Graph 1. QIDS scores of patient K.

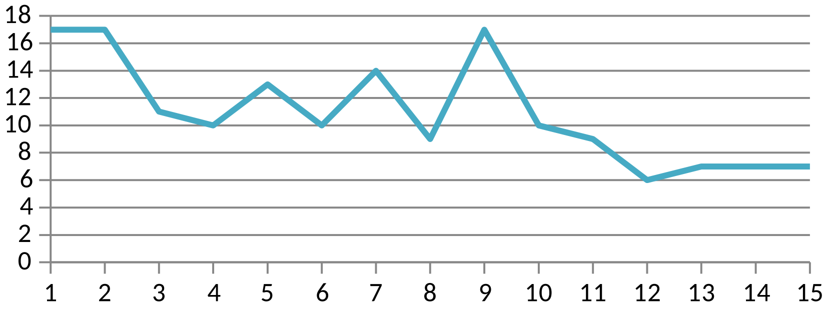

Table 1. Quotes and therapist behavior codes of online sessions of patient K.

Online sessions	Quotes from feedback messages	Codes
Online session 2 'Clarifying your symptoms'	Feeling guilty, is that something you always do (to a greater or lesser extent)? Or do you suffer from it now, during your depression? (In any case, it is also one of the symptoms of depression, but it can also be something that you do anyway, and then it is probably a long-standing thought pattern.	asking questions to clarify, psychoeducation
Online session 3 'Motivation and setting goals'	We have just discussed that you will repeat this session again. You will briefly describe the day, so that you can get concrete goals for the coming period. What do you want to achieve with the therapy? Make that concrete and think about one (or more) steps that you can take. We just discussed that it might be wise, for example, that you give your child more responsibility. You do not have to do everything for her. Children are allowed to do tasks, that is also part of parenting.	summarizing f2f, asking 'thought' questions, giving suggestions, normalizing
Online session 10 'Looking at the future'	You have achieved good results with the therapy. You can enjoy time with your child and friends again and you realize how this happened by structurally taking too much hay on your fork. You now know how it feels to be rested. You have gained many insights by critically examining your own thoughts. Keep challenging your thoughts, so that your new thoughts / rules of life will become increasingly credible. Your score on QIDS = 7, so still mild complaints (0-5 = no depressive symptoms). This means that you experience a lot of improvement, the	summarizing online, inciting future behavior, informing about the monitoring, validating/interpreting

symptoms you have are still light and I expect if you persevere, that you can maintain the improvement

Box 2. Case description of patient A.

Background information

A. is 25 years old and experiences problems with her work. Her symptoms have been there for two to three years now. She has a negative self-image and shows a significant amount of avoidance behavior. She sometimes thinks of suicide, but says she doesn't want to die. Her family lives in Turkey.

Treatment course

A. completed 8 online sessions (repeated online session 5) and had 5 face-to-face sessions with her therapist. At the beginning of treatment she scores 18 on the QIDS, which is considered severe. After 17 weeks of treatment A. is still experiencing severe symptoms of depression and she scores 21 on the QIDS. Despite this, she indicates at the end of treatment that she has gained a lot of insight from the treatment. Apart from the online sessions, she also received guidance from a self-help book about a negative self-image. After treatment she wants to continue to use the online thought-schemes. Most used therapist behaviors were Informing (relative frequency 31.6), Affirming (relative frequency 21.1) and Encouraging (relative frequency 19.3).

Graph 2. QIDS scores of patient A.

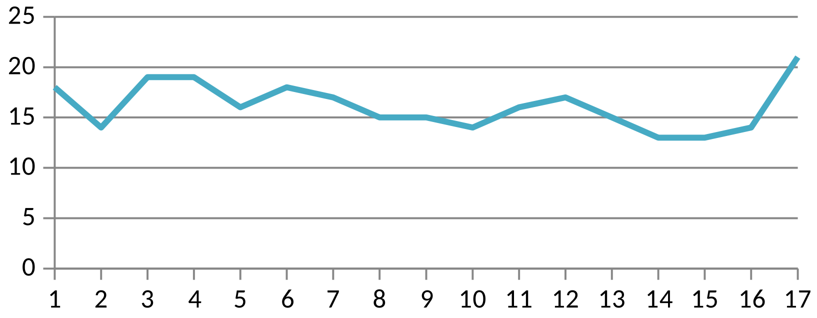

Table 2. Quotes and therapist behavior codes of online sessions of patient A.

Online sessions	Quotes from feedback messages	Codes
Online session 2 'Clarifying your symptoms'	Very nice that you managed to go through the first session and that you succeeded in completing the exercise. Good to hear that you have experienced it as helpful to put everything in order. Good that you filled in the questionnaires! We will discuss them later. I see that you also kept the diary for one day. It is important to do this consistently so that we get an idea of what the link is between your activities and the symptoms you have.	praising past behavior, informing about the monitoring, diary, urging
Online session 5 'Pleasure and fulfilment'	I see that thinking of enjoyable activities goes well, but that you find it difficult to implement them. We have already discussed this in the face to face sessions several times. It's good that you've tried it. You say that you want to do the session again. Shall we agree that you will do it one more time and then actually carry out the intended activity? Try to think of something that is easy to carry out and that cannot go wrong. Whatever happens, allow yourself this activity. Maybe you can also think of a way to reward yourself when you succeed?	validating/interpreting, summarizing f2f, praising past behavior, giving suggestions, urging, asking 'thought' questions
Online session 6 'Structure and planning'	You indicated earlier that you benefited from previous structures and planning for work. So it is worth to try to incorporate this in your private activities as much as possible as well. Try to make a planning for each day by using the steps: Step 1: Plan your every-	summarizing f2f, informing about the assignments, giving suggestions, inciting future behavior

day activities, Step 2: Plan your must-activities, Step 3: Plan your enjoyable activities. Good luck with the next module about the thought-scheme!

Box 3. Case description of patient M.

Background information

M. is 25 years old. His depressive symptoms are associated with emotional neglect by his parents. M. is very driven and ambitious, in combination with a vulnerable and low self-esteem. He supports his parents wherever he can, especially financially, but doesn't get recognition for this. M. also suffers from concentration and reading problems following an accident.

Treatment course

M. completed 4 online sessions and had 8 face-to-face sessions with his therapist. At the beginning of treatment he scores 19 on the QIDS, which is considered severe. At the last known QIDS he scores 11. M. indicates that the therapy has helped him to get more insight into the dynamics within de family. He feels less responsible for his parents. His mood has improved. His self-image and knowledge about his needs remain diffuse. Most used therapist behaviors were Encouraging (relative frequency 28.6), Affirming (relative frequency 21.4) and Questions (relative frequency 21.4).

Graph 3. QIDS scores of patient A.

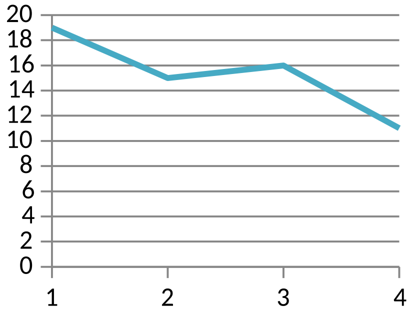

Table 3. Quotes and therapist behavior codes of online sessions of patient A.

Online sessions	Quotes from feedback messages	Codes
Online session 2 'Clarifying your symptoms'	I see you've been working well on the assignment. What was it like to write about your depressive symptoms? It is indeed difficult if you are not in a good mood to do things. And that if you manage to do things, that this will have a positive influence on your mood.	praising past behavior, asking 'thought' questions, validating/interpreting
Online session 3 'Motivation and setting goals'	Thank you for completing the assignments. Well done. You write that is the first step is very difficult, since you want to be less busy, but that means more free time and peace and that turns into thinking and worrying. But is this then a good reason not to take a rest? What could you do about the rumination?	praising past behavior, summarizing online, confronting, asking 'thought' questions
Online session 4 'How active are you?'	Good that you have found a way to deal with this exercise. Has it helped to get better insight between the relationship of activity and mood? You can find more helpful questions in the assignment. Good luck.	praising past behavior, asking questions to clarify, informing about the assignments, inciting future behavior
